# Unpacking the Oral Healthcare Landscape in India: A Qualitative Inquiry into Strengths, Shortfalls, and Future Directions Through the Lens of Public Health Dentists

**DOI:** 10.3390/ijerph22111741

**Published:** 2025-11-18

**Authors:** Parul Dasson Bajaj, Ramya Shenoy, Latha Davda, Kundabala Mala, Gagan Bajaj, Ashwini Rao, Navya Karkera, Srinivas Pachava, Mithun Pai, Praveen Jodalli, Avinash Badekkila Ramachandra

**Affiliations:** 1 Department of Public Health Dentistry, Manipal College of Dental Sciences Mangalore, Manipal Academy of Higher Education, Manipal 576104, India; parul.bajaj@learner.manipal.edu (P.D.B.); ashwini.rao@manipal.edu (A.R.); navya.k@manipal.edu (N.K.); mithun.pai@manipal.edu (M.P.); praveen.jodalli@manipal.edu (P.J.); avinash.br@manipal.edu (A.B.R.); 2 Civilian Dental Surgeon, Ministry of Defense UK and Adjunct Faculty, Manipal College of Dental Sciences Mangalore, Manipal Academy of Higher Education, Manipal 576104, India; davdalatha@gmail.com; 3 Department of Conservative Dentistry and Endodontics, Manipal College of Dental Sciences Mangalore, Manipal Academy of Higher Education, Manipal 576104, India; kunda.kamath@manipal.edu; 4 Department of Audiology and Speech Language Pathology, Kasturba Medical College Mangalore, Manipal Academy of Higher Education, Manipal 576104, India; gagan.bajaj@manipal.edu; 5 Department of Public Health Dentistry, Sibar Institute of Dental Sciences, Takkellapadu, Guntur 522509, India; srinivaspachava@gmail.com

**Keywords:** oral health, public dental health, universal healthcare, India, qualitative research, oral health services accessibility, oral health policies

## Abstract

The World Health Organization’s Bangkok Declaration, ‘No health without oral health,’ recognizes oral health as a global public health priority. Despite being largely preventable, oral diseases affect nearly half of the global population, and India mirrors this crisis while facing persistent systemic challenges. This qualitative study explores India’s oral healthcare landscape from the perspective of public health dentists to inform context-sensitive reforms. Thirty-one in-depth interviews were conducted with public health dentists from dental colleges registered with the Dental Council of India, recruited across six regions. Interviews were conducted online via MS Teams using a piloted interview guide and video-recorded with consent. Subsequently, the interviews were transcribed verbatim, anonymized, and qualitative data was analyzed using Atlas.ti, following reflexive thematic analysis. Analysis yielded four main themes: facets of oral health inequalities, dental public health initiatives, strategies to mobilize and optimize dental workforce in rural areas, and recommendations to optimize oral healthcare. This study offers contextually grounded yet globally relevant perspectives on oral health reform. By bridging local insights with international priorities, this study proposes a sustainable, equity-driven framework for transforming oral health systems while laying the foundation for future research and policy action aimed at achieving universal oral health coverage.

## 1. Introduction

A significant paradox in global health is that, despite being largely preventable, the combined prevalence of oral diseases is higher than that of other non-communicable diseases (NCDs) worldwide [1]. Among approximately 3.7 billion people, or nearly half the global population, untreated oral diseases significantly diminish the quality of life through pain, functional limitations, disfigurement, and in severe cases, even death [2,3]. Moreover, the economic impact of oral disease cannot be overlooked, with global expenditures amounting to nearly 387 billion US dollars (USD) in direct costs and 323 billion USD in indirect costs as of 2019 [3]. These figures further reinforce the World Health Organization’s (WHO) Bangkok Declaration ‘No health without oral health’, which calls for making oral health a global public health priority by recognizing it as an important NCD and advancing toward Universal Oral Health Coverage [4].

India mirrors this global trend, with the Global Burden of Disease Study 2019 identifying it as the leading contributor to the rising burden of oral disorders in South Asia, with the largest increase in age-standardized disability-adjusted life-years (DALY) rates for oral diseases between 1990 and 2019 [5]. The burden is so substantial that India contributes to 18% of the global cases of dental caries in both permanent and deciduous teeth, 20% of severe periodontal disease cases, and 23% of lip and oral cancer cases globally [6]. This growing burden is primarily driven by systemic inequalities that manifest across multiple levels. At the individual level, barriers such as fear of dental procedures, limited awareness, and poor oral health-seeking behavior hinder access to care. Socio-demographic inequalities further compound the problem, disproportionately affecting children, the elderly, and gender minorities, with geographic and economic divides intensifying these challenges, with rural communities and individuals from lower socioeconomic backgrounds facing the greatest disadvantages [7]. These challenges are magnified by the structure of dental care delivery, where most patients are required to pay out-of-pocket (OOP) for services, whether in public or private settings, with heavy reliance on the private sector [8,9].

These inequalities thereby hinder access to essential oral healthcare, which includes a set of safe, quality, and cost-effective interventions such as regular oral health screening, delivered at both individual and community levels to promote oral health, as well as prevent and treat the most prevalent oral health conditions, while also ensuring appropriate rehabilitation and referral where needed [10,11]. Access to oral healthcare is especially limited in rural areas, where fewer dentists practice due to the concentration of professionals in urban centers seeking better financial opportunities [7]. Additionally, the absence of comprehensive, targeted oral health policies along with low budget allocation for oral health continues to exacerbate this issue [5,9]. This aligns with the suggestions from a recent scoping review that emphasized the need to shift from treatment-focused approaches to preventive, inclusive, and integrated oral healthcare, supported by stronger policies, increased awareness, and cross-sector collaboration [7].

This shift requires the adoption of models that are responsive to India’s rich sociocultural and regional diversity, alongside policy reforms grounded in a deeper understanding of the complexities within the existing oral healthcare system. This aligns with the growing recognition of the need to develop population-specific models that reflect both regional and national contexts [5]. Crucially, these efforts must be informed by the voices of key stakeholders such as patients, healthcare providers, and policymakers, whose viewpoints and lived experiences offer valuable insights into the challenges and opportunities within the oral health system [12]. In this context, qualitative research offers a powerful lens through which these perspectives can be explored, capturing the lived experiences and professional judgments that are often underrepresented in policy discourse [7,12].

Building on this foundation, this qualitative research was designed to explore the perspectives of public health dentists across India through in-depth interviews. This study focused on understanding the current oral healthcare landscape and examining the potential role of dental health associate professionals (DHAPs) [13], such as dental hygienists, dental therapists and dental nurses, in addressing oral health inequalities in India. Given the breadth and complexity of the overall research, the present paper primarily aims to illuminate the current state of oral healthcare in India, as articulated through these in-depth interviews. Specifically, this paper addresses two objectives: (1) identifying the strengths and shortcomings of the existing oral healthcare system in India and (2) exploring potential pathways for optimizing India’s existing oral health system. The findings from this study could inform broader policy implications and serve as a foundation for our subsequent paper on the prospective utilization of DHAPs in the Indian oral healthcare system.

## 2. Materials and Methods

### 2.1. Study Design and Context

This qualitative study is presented in compliance with the Standards for Reporting Qualitative Research (SRQR) guidelines [14], as detailed in Supplementary Material S1. Ethical approval for the study was obtained from the Institutional Ethics Committee (22057). This study is grounded in critical realism as its ontological framework and adopts contextualism as its epistemological position, acknowledging the existence of an objective reality while recognizing the plurality of interpretations based on subjective experiences within specific sociocultural and professional contexts [15].

### 2.2. Study Participants and Sampling Strategy

The study participants were public health dentists holding academic positions at dental institutes across India. India has about 1600 public health dentists with master’s training, equipped to address community oral health needs through health education, promotion, prevention, research, and policy development. Public health dentists in academic roles regularly lead community programs, supervise student outreach, and work with local health authorities in underserved areas. These experiences provide them with practical insight into the existing challenges of the population, making them suitable participants for this qualitative study [16]. For this qualitative study, a purposive sampling approach was employed to capture diverse perspectives and ensure heterogeneity among participants. The participants were selected from those previously recruited for the quantitative phase of the broader mixed-methods research and expressed a willingness to participate in an in-depth interview. While the selection was purposive, the distribution of interviews across the six regions of India was kept nearly proportional to the number of dental colleges within each region in the quantitative sample [17]. This approach allowed for both diversity in viewpoints and balanced regional representation. The interviews were concluded upon achieving overall saturation in responses during the data collection phase, when no new information emerged from participants’ responses, resulting in a total of 31 in-depth interviews.

### 2.3. Data Collection

The interview guide was developed by combining the conceptual model for personnel mix developed by Buchan et al. [18] with the findings of the scoping review [7] on oral health inequalities in India. A piloted interview guide (Table 1) was used to conduct in-depth interviews online via the Microsoft Teams platform. The guide was iteratively refined during the data collection process, incorporating additional lines of inquiry to further explore region-specific challenges in accessing oral healthcare. Prior to each interview, participants’ consent for video recording was obtained, and informed consent was recorded. The interviews were conducted in English by the primary researcher (PDB) between January 2024 and June 2024, with each interview lasting between 30 and 60 min.

The in-depth interviews were conducted by the primary researcher (PDB), a female doctoral candidate with a master’s degree in orthodontics and formal training in qualitative research methods through university-verified doctoral coursework. Given that the participants held faculty positions in public health dentistry, they occupied a position of authority and professional privilege during the interviews. In contrast, the interviewer, being an orthodontist and relatively new to the field of public health dentistry, maintained a neutral stance, approaching discussions with openness and minimizing potential biases.

### 2.4. Data Processing and Analysis

The recordings of the in-depth interviews, along with the automated transcripts generated from Microsoft Teams, were securely stored in password-protected folders. One researcher (PDB) carefully listened to the interview recordings multiple times to transcribe them verbatim, ensuring accuracy by reviewing and refining the automated Microsoft Teams transcript for errors and inconsistencies. All transcripts were anonymized during the transcription process to ensure participant confidentiality.

Reflexive thematic analysis was used to analyze the data, enabling inductive coding across a broad spectrum of semantic and latent levels while providing the flexibility to navigate the data along a continuum from descriptive observations to deeper interpretative meaning-making [15]. The analysis was carried out between May 2024 and February 2025 via Atlas.ti 24.1.0 for Mac [19]. The initial coding was undertaken by one researcher (PDB), following which they were reviewed by a second researcher (RS), leading to the finalization of the codes. These finalized codes were then reviewed to identify and develop themes that closely aligned with the research objectives. These themes were subsequently reviewed by two researchers (RS and LD) independently to ensure their accuracy and analytical rigor. Ultimately, the findings were organized into overarching themes, main themes, and sub-themes. Throughout the process, the primary researcher (PDB), as an outsider to public health dentistry, maintained neutrality and reflected on their thoughts through reflexive journaling. Regular engagement with relevant literature, along with frequent discussions of data interpretations with co-researchers from the field of public health dentistry, further enhanced contextual understanding and analytical balance, thereby supporting the credibility of the findings.

### 2.5. Trustworthiness

Various methods, such as data familiarization, member checking, triangulation, and peer debriefing, were employed to enhance the trustworthiness of this research and ensure methodological rigor [20,21,22]. The primary researcher (PDB) maintained prolonged engagement from the outset by conducting interviews lasting between 30 and 60 min, followed by persistent observation through multiple interactions with the data. Member checking was performed during the interviews by summarizing the responses and confirming them with the participants in real time. Researcher triangulation involved two additional researchers (RS and LD), while data triangulation was achieved by corroborating the findings with a prior scoping review on oral health inequalities in India [7]. Detailed documentation of methods ensured dependability, while peer debriefings helped minimize bias, and an audit trail was maintained to promote transparency and confirmability.

## 3. Results

In this qualitative research, 31 in-depth interviews were conducted with public health dentists holding academic positions in dental colleges across six regions of India, with the distribution details presented in Table 2. The analysis of this qualitative study identified several insights into the oral healthcare landscape in India, which are presented in this paper under four main themes namely, facets of oral health inequalities, dental public health initiatives, strategies to mobilize and optimize dental workforce in rural areas, and recommendations to optimize oral healthcare. These themes are discussed in detail in the subsequent sections.

### 3.1. Facets of Oral Health Inequalities

This first main theme offers insights into the foundational challenges shaping the oral healthcare landscape in the Indian context, with its breadth covered across five sub-themes, namely population variants, regional variants, dental education variants, dental workforce and delivery variants, and policy variants.

#### 3.1.1. Population Variants

The first sub-theme, population variants, is based on participants’ reflections on population groups experiencing inequalities, along with the attributes that may hinder their access to oral healthcare services. The participants identified children, older adults, the rural population, and tribal groups, among others, as the most affected.

*P1: “The worst part of dental care is that extremes of age, like children on one side and geriatric population on the other side… are affected.”*

*P24: “… I think the bitter truth is, the rural population is still devoid of proper health care and there are reasons to it because there is a dearth of qualified dentists in rural area…”*

*P10: “Like I said before, we work with tribal people, in such areas, the access towards dental healthcare has been less.”*

The participants noted that lower prioritization of oral health compared to general health, coupled with a lack of awareness and limited oral health-seeking behaviour, were key attributes hindering access to oral healthcare services. These challenges were further intensified by barriers related to affordability and accessibility.

*P4: “…from both the government side and from the individual side that kind of importance is just not given to oral health… The disease burden is too much, and that kind of attention and resources are not diverted to oral health.”*

*P26: “I feel people are really short of oral care resources and are not able to access it, they have financial issues, resource related crunches, distance related issues and so on…”*

#### 3.1.2. Regional Variants

The second sub-theme, regional variants, collates regional concerns raised by the participants, highlighting specific oral health challenges across different parts of India.

Northeastern and Eastern regions: Participants reported a general lack of awareness about oral health in these regions. These areas were also identified for higher prevalence of tobacco use, especially smokeless tobacco, and consequently, a higher incidence of oral cancer.

*P8: “So, the tobacco consumption is very high here especially smokeless tobacco. So not only tobacco oral hygiene is very poor.”*

Additionally, the participants highlighted the weather-related challenges as well as cultural and language barriers specific to certain populations, such as tribal communities, as important factors in the provision of services in the northeastern region.

*P8: “So tribal population is more here, so I feel like the cultural differences, the language barrier also comes into account…”*

*P8: “And what I feel most of the districts are flood affected, so people see if in a neighbouring district they find the dentist…”*

Western region: Similar challenges were echoed by a participant from the western region, particularly concerning oral health awareness among tribal communities and the prevalent use of smokeless tobacco.

*P4: “But we’ve had experiences here with tribal population… I mean, in a child’s mouth, the mother puts tobacco from the time the child is 2–3 years old. There was a settlement of about 250- 300 tribal people and almost everybody was using tobacco…”*

Central and Southern regions: Participants from southern and central regions also shared similar observations, noting that despite awareness efforts and tobacco control measures, the use of smokeless tobacco remains widespread, contributing to a continued high burden of oral cancer.

*P23: “I feel the burden of oral cancer is high… on one end we provide a lot of awareness but on the other end, we get a high number of oral cancer cases…”*

*P28: “Though there are so many rules and regulations regarding tobacco use and in spite of all the efforts by the tobacco health programmes. The common man still is very more prone for the tobacco use, especially smokeless.”*

#### 3.1.3. Dental Education Variants

Dental education variants, the third sub-theme, focuses on participants’ insights into the shortcomings of the dental education system. These include the concentration of dental institutes around urban areas, gaps in the existing dental curriculum and a lack of awareness among dental students regarding the hardships faced by underserved populations in accessing dental care. Specifically, in the northeastern region, there was an opinion that there are too few dental colleges and an insufficient number of postgraduate seats available.

*P6: “Everybody wants to set up their practise or even academic institution (are in urban areas), very few are located in the rural areas. And so that discrepancy till today is there…”*

*P16: “Our undergraduate teaching methodologies and curriculum needs to be revamped and showcased in a new manner… because our curriculum is totally focused on curative (operative) services…”*

*P15: “We are going for camps (to treat people in rural areas) and many people are there who cannot leave their one-day income and come to college because even if you come to college, the students are really unaware of what struggles they have undergone (to come to the city).”*

#### 3.1.4. Dental Workforce and Delivery Variants

The fourth sub-theme, dental workforce and delivery variants, emphasizes the challenges encountered in ensuring that dentists and dental care services effectively reach the population. Participants suggested that the issue was not the number of dental professionals but their uneven distribution and lack of alignment with regions having a greater oral disease burden. This maldistribution, particularly between rural and urban areas, is further exaggerated by factors like limited job opportunities, which drive the dental graduates to migrate abroad or pursue alternate careers, and attrition among female dentists due to domestic and caregiving responsibilities.

*P16: “… there are a lot of dental colleges. There are a lot of graduates being produced. The burden is also high. Manpower is also high. The problem is that the manpower and the burden are not in sync.”*

*P1: “Yeah, see what is happening is that 70% of rural area in India and hardly any dentists are there at all.”*

*P8: “… there’s very few job opportunities for the dental graduates.”*

*P16: “… how many dentists are there who are into active practise or for that matter to academic job. I think we’ll have shocking numbers because many dentists either have flown out of the country or many dentists have just given it up and they are living a homemaker’s life or something else.”*

Upon further discussion, the participants stressed that dental manpower is predominantly concentrated in urban areas, as dentists are often reluctant to work in rural regions because of inadequate compensation and a lack of essential infrastructure, such as roads, schools, and other facilities necessary for relocating their families. Additionally, participants noted that financial concerns hindered the establishment of dental clinics in rural areas, as recovering the initial investment posed a significant challenge.

*P10: “Most of the practitioners, uh tend to prefer urban area for their practise because we all know that setting up a dental practise is quite expensive nowadays… urban areas where the chance of getting returns much higher and settling in urban area also offers other advantages as well in terms of their family and schooling and things like that.”*

With respect to oral healthcare delivery in India, participants highlighted its heavy reliance on the private sector, resulting in high costs for patients. This financial burden limits accessibility and creates disparities in service provision.

*P18: “In the private sector, the whole problem is the out of pocket expenditure for the dental treatments that’s happening and 80% of the services are being provided by the private sector and only 20 or even less, I would say by the public sector.”*

Other challenges included resource limitations, financial constraints, and shortages of materials and equipment. These challenges resonated not only in the field settings but also across both government and private dental healthcare setups, as reflected in the following excerpt.

*P25: “When a Primary Health centre (is set up) where a medical professional is there and a dentist is there, until or unless it’s like we are handicap, if we don’t have those equipment or chairs and the materials right… because setting up of a dental clinic in rural areas is a very costly affair, very costly affair for even the government.”*

#### 3.1.5. Policy Variants

Participants also raised concerns regarding the deficiencies in the implementation of the oral health policy and the absence of well-defined regulations within the dental profession, giving rise to the fifth sub-theme, policy variants. In addition, participants also emphasized on inadequate budget allocation for oral healthcare and the limited availability of job opportunities for dentists within primary healthcare settings.

*P7: “I mean to say oral health policy is not in place… And also we do not have sufficient budget for oral healthcare and it (oral health) is then most often neglected.”*

*P9: “… even if you see in the remote area in primary healthcare… they don’t have that dental part there, they don’t have appointment of dentists in primary healthcare.”*

### 3.2. Dental Public Health Initiatives

The second main theme, dental public health initiatives, focuses on the positive aspects of the existing oral healthcare system in India and can be divided into two sub-themes of community-based initiatives in dental colleges and positive trends in Indian oral healthcare.

#### 3.2.1. Community-Based Initiatives in Dental Colleges

The first sub-theme, community-based initiatives in dental colleges, emerged from discussions with the participants about their work in the field. It highlighted diverse activities and services provided by public health dentists in dental colleges, who actively engaged with communities through awareness programs, community-based dental education and research. Their work included a broad range of services such as oral health education, disease screening, including oral cancer screening, preventive procedures, comprehensive treatment, and tobacco cessation counselling. These services were made accessible through outreach camps, peripheral centers, mobile dental vans, and portable chairs, as well as through collaboration with primary health centers, non-governmental organizations (NGOs), and government bodies.

In addition to these foundational efforts, more targeted interventions included adopting villages and schools, implementing specialized programs for tribal communities and specially abled children, and providing palliative care. They also played a role in capacity-building initiatives which focused on training teachers, accredited social health activists (ASHAs), and Anganwadi workers, and empowering caregivers of vulnerable populations. Moreover, these public health dentists contribute to policy implementation, particularly through their involvement with the National Oral Health Policy and the Clinical Establishment Act. Collectively, these efforts are an integral part of the role that dental colleges play in delivering community-based oral healthcare, addressing disparities, and fostering a more inclusive approach to dental health services.

*P13: “Based on the needs, we are providing them the service in the field,… includes health education, health promotion and some of the basic services (such as) prophylaxis, fillings or extractions and then we refer them for further treatment to the institute.”*

*P4: “we also got funding from National Health Mission, National Oral health programme and we have carried out teachers training programme, pit and fissure sealant application programme.”*

#### 3.2.2. Positive Trends in Indian Oral Healthcare

The second sub-theme, positive trends in Indian oral healthcare, emerged from participants’ acknowledgement of the gradual progress in improving dental care access and policy implementation, which they viewed as small yet meaningful steps forward. At the policy level, they appreciated the growing momentum towards the National Oral Health Policy (NOHP) and the integration of oral health into broader national health initiatives, which has increased awareness among both the public and policymakers.

*P18: “the programme (National Oral Health Programme) was launched in 2014 and 2015… People have identified that national oral health programme is a programme that’s being implemented and along with age-old programmes like NTCP (National Tobacco Control Programme), RNTCP (Revised National Tuberculosis Control Programme), mother and child program, oral health is also one among them. So, I would say maybe our visibility has increased.”*

Additionally, participants noted that the deployment of dentists in National Health Programs and the expansion of rural clinics, particularly in the North East, has further enhanced access to dental care. The state of Kerala (south) stood out in the interviews for its strong oral healthcare network, attributed to better road connectivity, which has improved oral care access and outcomes in rural areas.

*P6: “Kerala is very different, we do have remote areas as in other states, but we are well connected (road connectivity) that even if you say it is a rural area in Kerala, you’ll find that all the facilities are there, including dental clinics.”*

### 3.3. Strategies to Mobilize and Optimize Dental Workforce in Rural Areas

As outlined in this third main theme, the interviews also explored alternative strategies to channelize the existing dental workforce into the rural areas. Participant perspectives that emerged have been organized into three sub-themes, namely dental education reforms, government-led initiatives and research and strategic partnerships.

#### 3.3.1. Dental Education Reforms

One of the primary recommendations from participants in dental education reforms was the implementation of compulsory rural postings for dental graduates to increase access to oral healthcare in underserved regions.

*P7: “… students should have a compulsory rural posting… for one month if we make the students stay there, you know, then they will have an idea of how life is in rural areas, what is the oral health status of people there and what is their lifestyle. The students may get motivated to work at this ground level and help them out.”*

Additionally, the participants expressed the need to align dental education with societal needs while restructuring the dental curriculum to cultivate a community-driven, service-oriented mindset from the very beginning, thereby positively shaping dental students’ perceptions of public health services.

*P25: “Proper curriculum related to community based dental education has to be there, where all the social, behavioural concepts and psychological concepts have to be taken into picture and the approach towards it, and when the field work is done by the students, reports are generated out from the work done by these students, recommendations and feedbacks are sought from them., That’s what I’m telling that community based dental education is a big concept which actually needs to be worked out again and implemented in India for the dental curriculum.”*

Participants also spoke about the Sevagram initiative under the National Dental Commission, which proposes a family adoption model in which dental students take responsibility for the oral healthcare of assigned families throughout their training, as reflected in the following excerpt.

*P24: “the National Dental Commission’s proposal for introduction of sevagram model from first year. And in that each student from first year will be allotted a house in whichever slum the college chooses, and that first-year student will be looking after the oral health of all the family members of that house from first year till his internship.”*

Additionally, one participant suggested establishing new dental colleges in underserved rural regions and encouraging greater enrolment of students from rural backgrounds to study dentistry, thereby cultivating a workforce more likely to serve their communities.

*P7: “But you know setting up a dental College in rural area, then encouraging the youngsters who have passed out from government schools and junior colleges there to actually get admitted for Bachelor of Dental Surgery (BDS) courses… They might set up their own practice there.”*

#### 3.3.2. Government-Led Initiatives

The second sub-theme, government-led initiatives consolidates participants’ suggestions on the measures the government could implement to address the skewed distribution of dentists in the rural areas within India. Participants felt that to create sustainable employment opportunities, the government should prioritize rural placements by incorporating dentists into primary healthcare centers (PHCs) and community health centers (CHCs) as well as in National Health Programs. Additionally, they highlighted the need for better remuneration and enhanced support for dentists working in the public sector.

*P19: “… job opportunities for dentists in public sector need to be increased. And I think another way to attract the dentist to work in a rural setup would be the incentives, such as the salaries are high compared to a private setup… good residential quarter with good amenities. All of this would you know, kind of motivate them to leave their base at the urban area and probably do justice to their jobs in their rural postings.”*

*P8: “… dentists have been incorporated into this Rashtra Bal Suraksha Yojana for maternal and child health. Yeah. If National Health programmes incorporate dentists… is a great initiative by the government.”*

Participants also suggested establishing Public–Private partnership (PPP) models as a strategic approach to enhance service delivery and improve rural dental care by bridging the gaps in infrastructure, funding, and manpower through collaboration between the government and private entities.

*P25: “And again, PPP (Public Private Partnership) model… we should harness that, even the pharmacy sector, where as a part of social responsibility they can do lot of things in terms of rural areas…”*

Apart from creating job opportunities in the government sector, the participants suggested that the government could offer financial assistance to dentists who are currently running or planning to establish rural dental practices. This support could include interest-free or low-interest loans for setting up rural clinics, along with subsidized equipment, materials, and even free electricity to help minimize operational costs. Additionally, participants proposed that the government could partner with existing private dental clinics through a pay-per-patient model to improve access to affordable dental care for rural populations.

*P5: “So you know, maybe some incentives can be announced for people who are planning to open up clinics in rural areas, maybe like, you know, if they want some loans, maybe the loans can be given at a lower rate of interest and you know, maybe machinery, the equipment can be provided to them at subsidised rates.”*

#### 3.3.3. Research and Strategic Partnerships

Participants also proposed other divergent strategies to channelize workforce, which led to the emergence of the third sub-theme, research and strategic partnerships. They emphasized the need to conduct research to systematically identify the barriers preventing dentists from establishing practices in underserved rural areas and to develop targeted strategies for addressing these challenges, as observed in the following excerpt.

*P6: “And we should try to address the problems as to why till date dentists don’t want to go set up in a rural area. So, you know there will be people who are interested probably, there you should find out what exactly are their limitations and why they don’t want to set it (dental clinic) up there even if they are inclined.”*

In order to mitigate the financial constraints associated with establishing and maintaining dental practices in rural regions, participants suggested leveraging corporate social responsibility funds to support dental practitioners. Additionally, NGOs were identified as potential facilitators in complementing governmental initiatives by providing financial assistance and operational support, thereby fostering a more sustainable and effective rural dental healthcare system.

*P11: “Government should create a channel like that so that these dentists (dentists with clinics in rural areas) can get help. It may not be through the government directly—maybe through an NGO (Non-Government Organization) or through a CSR (Corporate Social Responsibility) fund, something like that. There should be a proper setup for this so that dentists will also be happy working there, because you cannot charge all these underprivileged people so much money just to recover the initial investment.”*

### 3.4. Recommendations to Optimize Oral Healthcare

The fourth main theme, recommendations to optimize oral healthcare emerged as an offshoot of the discussions with participants during the interviews and brought together a set of strategic suggestions to improve accessibility, efficiency, and the overall quality of care. These include dental education measures, downstream measures and upstream measures.

#### 3.4.1. Dental Education Measures

Dental education measures focus on optimizing oral healthcare through changes in dental education. Among the various recommendations, participants emphasized the need to update the existing dental curriculum to strengthen clinical skills among dental graduates and to move toward a more comprehensive model of dental care. Additionally, they recommended incorporating administrative knowledge to equip dentists with the skills needed to manage healthcare organizations.

*P25: “I think students need to know how organisations are run, engage them more… lot of collaborations should be made with organisations, maybe short internship or something, they can be rotated in those department or in those dispensaries, wherever they are put… and maybe they can do a structured interview with a person who’s in charge over there and short feedbacks related to that, from patients to the top-level in that setup only.”*

Participants highlighted the need for a coordinated effort among all dental colleges to improve access to oral healthcare in rural areas. They recommended that dental institutions should work collaboratively and partner with the government with the aim of making a wider impact within the community. Participants believed that the dental colleges should take an active role in primary healthcare by overseeing PHCs, with each college assigned to a district or a group of PHCs within a taluk, thereby increasing accessibility and ensuring equitable coverage across the country.

#### 3.4.2. Downstream Measures

The second sub-theme, downstream measures, focuses on actionable steps to enhance oral health by engaging communities at the grassroots level. A key recommendation from participants was to raise awareness among the general public and healthcare providers, particularly through social media, by using culturally appropriate programs designed to reach people from diverse social and economic backgrounds.

*P21: “Awareness still needs to be created though it is there in the urban areas, rural areas still need to be concentrated, and I think it is essential to tailor the message locally rather than projecting it in the national or an international forum… I think it should be locally and culturally acceptable.”*

An interesting concept that emerged during the discussion on awareness was the idea of “putting the mouth back into the body,” highlighted by one participant (P18). This concept went a step further than the widely recommended integration of oral health into general health during the interviews. These findings suggest that dentistry should not be treated as a completely separate field, but rather as a branch of medicine, so that oral health receives the importance it truly deserves.

*P18: “There is a school of thought which also thinks that the mouth should be put back in the body for getting its due importance. The training (for medicine and dentistry) should be common and then when you have your specialisations, you segregate that time.”*

Participants also recommended adopting a preventive approach, particularly by targeting children through early detection of dental diseases and proposed a gradual shift towards incremental care that prioritizes prevention over curative treatments. Additionally, they believed that access to oral healthcare could be improved through the use of mobile dental units, tele dentistry, and portable dental chairs, as these solutions reduce infrastructure costs in rural areas and allow for more efficient and flexible deployment of dental professionals across various locations. In addition, participants felt that setting up satellite clinics for underserved populations and organizing structured weekly or monthly programs targeted at specific groups could support broader and more sustainable access to dental care.

#### 3.4.3. Upstream Measures

Upstream measures, the third sub-theme, offers a multifaceted approach to strengthening oral health systems and policies across the country through targeted, systemic reforms. Among the recommendations, a prominent insight was the participants’ unequivocal recommendation to prioritize the implementation of the NOHP.

*P16: “… I’ll go back to the national oral health policy, wherein once it comes in, there will be some learnings from the first document and then I think the policy is going to organize the overall sector more.”*

Participants believed that oral healthcare could be significantly strengthened by drawing on the successes of existing healthcare models and national health programs through the integration of oral health components into their framework. Participants also emphasized that leveraging the well-established primary healthcare system to incorporate dentists would help ensure a comprehensive, equitable, and effective approach to oral health delivery nationwide.

*P18: “Oral health is a very new program, and I think that, thanks to all other old programs which has been running all this while, if we take learnings from these programs. But since we’ve talked about incentivization, the national blindness control programme came to my mind and all the cataract surgeries that were going on, it is not that all the surgeries were being performed by ophthalmologist who were there… there were NGOs that were involved and they roped in the private practitioners and then they incentivized each cataract surgery… Similarly, I think if we bring on more bodies or even directly incentivize the dentist, so maybe a state nodal officer or through a public programme. These efforts of a public system could facilitate dental surgeons into a rural area to work in a private setup.”*

*P18: “I’m saying that there are so many programs which require lateral integration. For example, there should be an oral health component to the mother and child programme. There should be an oral health component for the National Tobacco Control Programme and similarly for fluoride, the NPPCF, the National Programme for Prevention and Control of Fluorosis. Each programme should have an oral health component.”*

*P11: “Incorporating oral health into the Primary Health sector is the best way because India has such a nice healthcare system which is envied by, to be frank, by so many countries.”*

In addition, participants advocated for measures to enhance affordability, such as supporting local manufacturing of dental products to lower costs and introducing dental insurance to reduce the financial burden on the public.

*P21: “We have a proper insurance to represent oral health…… we can manufacture the dental products by ourself, the cost of the treatment would be brought down.”*

Finally, participants highlighted the need to reinforce the dental workforce by improving job opportunities and ensuring fair compensation for dental professionals, while also advocating for their representation in key administrative and policymaking roles. Additionally, they highlighted the need for the inclusion of dental auxiliaries in the dental workforce and the adoption of an integrated dental team approach to ensure effective oral care delivery across all levels of the health system.

*P18: “Each programme should have an oral health component. Maybe today they are manned by a medical officer, but later we understand that who better than a dentist would see such programmes? So down the line, a lot of dental positions should be created… This is where I feel midlevel dental fraternity can be added with equal dignity as any member of the medical team.”*

*P26: “I think there has to be a push for dental auxiliaries and dental auxiliary training, dental auxiliary training schools and recruitment of dental auxiliaries, it has to become a part of the National Oral Health Policy, then it will become a comprehensive national policy.”*

## 4. Discussion

This study offers valuable insights into the perspectives of public health dentists, illuminating the structural gaps and systemic inequalities that shape India’s oral healthcare landscape, along with the strengths, resilience and innovations emerging from within the system. The challenges and opportunities identified in the Indian context resonate with broader global patterns observed in oral healthcare systems, highlighting the universal nature of oral health issues. Reflecting this global trend, this qualitative research identified facets of oral health inequalities as a main theme, which is consistent with evidence showing similar disparities across various countries [23]. Significant inequalities have also been reported in high-income countries such as the United Kingdom (UK), the United States (US), and Canada [24] as well as in Japan, which is recognized for its high dental care utilization and minimal out-of-pocket (OOP) expenses [25]. These inequalities often manifest within specific socioeconomic groups and among vulnerable populations such as children, older adults, and individuals living in rural or remote areas, as observed in the present study and supported by literature from the US [26], Europe [27], India [7], Africa [28] and Asia [29].

A widely adopted strategy to improve oral health access globally is the implementation of targeted interventions for vulnerable populations [24,26,30]. Similarly, participants in the study advocated for focused strategies to reach underserved and geographically inaccessible communities such as establishing satellite clinics or conducting weekly or monthly outreach programs. Additionally, participants suggested the use of mobile dental clinics and portable dental chairs, an approach also embraced in Japan to serve its aging population [25].

Although these targeted efforts are essential for expanding access, they must be embedded within a broader, sustainable systemic transformation. This shift draws attention to a critical insight that emerged consistently across global discussions and participant interviews, the call to ‘put the mouth back into the body’ [31,32,33]. This powerful metaphor highlights the urgent need to integrate oral health into general healthcare systems and how the continued segregation of services perpetuates disparities in access and undermines the pursuit of holistic health outcomes [34]. Participants felt that recognizing oral health as an integral component of overall health could help strengthen its position within policy agendas, raise public awareness, encourage care-seeking behaviours, and enhance the utilization of existing dental services.

This vision aligns with current global health initiatives, including the WHO Global Oral Health Action Plan 2023–2030 and the Bangkok Declaration, both of which advocate the integration of oral health into universal health coverage (UHC) and broader NCD strategies [4,10]. Importantly, the holistic perspective of ‘putting the mouth back into the body’ must be embraced at multiple levels to drive integrated, meaningful and lasting change. These include the application of the common risk factor approach shared with other NCDs, the inclusion of dental coverage in general health insurance plans and most critically, the integration of oral health into primary healthcare systems globally.

A key starting point for this integration lies in the strong biological and behavioural link between oral health and other major NCDs through shared modifiable risk factors such as high sugar consumption, tobacco use, and alcohol intake [10,35,36]. These connections can be used to develop integrated strategies that not only enhance prevention but also improve cost-effectiveness across health systems. As suggested by the participants, this integration can be advanced in the Indian context by incorporating oral health into existing, well-established national health programs, thereby leveraging current resources and infrastructure. In addition to being a standalone concern, oral health can serve as both a reflection of systemic well-being and a gateway to the early detection of diseases that affect overall health [37,38]. A compelling example is Japan’s 2015 “New Orange Plan” for dementia care, which acknowledges the role that dentists could play in identifying patients with dementia, thereby contributing to earlier intervention and slowing disease progression [38].

However, these efforts towards integration require solid financial support, otherwise, the most well-integrated programs risk falling short of their potential [39]. Therefore, the second level of integration should involve financial mechanisms that support equitable access to oral healthcare. These financial mechanisms, developed by policymakers should support patients in accessing care through insurance coverage, subsidies, and other financing systems such as public health dental programs or third-party payment plans, thereby reducing financial barriers and improving service utilization. A pivotal step in this direction, as identified by the study participants, is the inclusion of dental services in general health insurance plans, thereby alleviating the high OOP expenditures associated with the heavy reliance on private dental care in India and promoting access for all, regardless of patients’ socioeconomic status. In India, the concept of comprehensive dental insurance is underdeveloped, with only a few health insurance policies offering limited dental coverage, often restricted to specific procedures [40,41]. In this context, the importance of insurance is further supported as evidence consistently suggests that high OOP expenditures negatively affect access to oral healthcare services [42].

International evidence reinforces the importance of strong public financing in improving oral care access. Japan’s well-established public insurance system provides extensive dental coverage along with medical treatment, resulting in one of the highest dental care utilization rates in the world and relatively low OOP expenses, averaging around 30% [25]. In contrast, OOP payments account for 60% of dental costs in Australia, largely due to comparatively low public investment in oral healthcare relative to other Organization for Economic Cooperation and Development (OECD) countries [31]. South East Asian countries present a similar picture, with Thailand reporting the lowest OOP expenditure (10.5%) followed by Indonesia, which is likely due to more established public health insurance schemes than other countries in the region [29]. These patterns suggest that OOP expenses are closely tied to the level of public subsidies and insurance coverage, which vary across countries based on health system priorities and funding.

Across Europe, public dental coverage models also vary widely with varying degrees of success in reducing inequalities. Universal coverage for children is available in Sweden, Germany, the UK, Denmark, Hungary, and Scotland; partial coverage exists in France and Ireland while Spain, Italy, and Greece provide little or no coverage [27,33]. For adults, substantial subsidies are provided in Germany, the UK, Sweden, and France, with Sweden delivering nearly half of adult dental services through its public health system. Meanwhile, adults in Greece, Italy, Romania, and Spain face high OOP costs due to limited public support and private insurance options. These variations are reflected in the utilization rates with approximately 75% of adults visiting a dentist annually in countries with well-funded systems like Germany and Sweden, compared with only 30–40% in underfunded countries such as Greece, Italy, and Romania [33]. Similarly, in the UK, where the National Health Service (NHS) ensures broad access to dental care, oral health inequalities are significantly lower compared to countries like the US and Canada where nearly one-third of the population lacks dental insurance [24]. Collectively, these models demonstrate how strong public investment in oral health can reduce financial barriers and promote higher utilization rates.

This brings us to the broader issue of how countries prioritize and fund oral health, which varies considerably across countries. High-income nations such as the US, Canada, the UK, and several European countries allocate substantial resources to oral health [24,27,33]. Among Southeast Asian countries, Thailand and Indonesia have some level of financing, although it remains limited, while the majority of countries in the region, including India, allocate only minimal funding for oral health [29]. This concern was even highlighted by the participants in this study, who felt that budget allocation for oral health in India is disproportionately low compared to other areas in healthcare funding. This underrepresentation, while reflecting on a lack of prioritization to oral health in national health agendas, highlights the need to adopt a more structured and equitable approach to oral health financing, one that draws on international best practices while addressing local realities.

Recognizing these international differences, it becomes imperative for policymakers to focus on strategic resource allocation and priority setting in oral health, a point also emphasized by participants in this study. Prioritization in oral health can take many forms, one area where resources could be directed involves financial coverage or subsidies in oral care for underserved populations, particularly in rural and remote regions where affordability and access remain major barriers. The recently proposed Canada Dental Care Plan is an example in this direction as it aims to extend dental coverage to uninsured Canadians with annual family incomes below 90,000 Canadian dollars (CAD) and no co-payments for those earning less than 70,000 CAD. Such an approach helps address challenges related to affordability, thereby supporting more equitable access to oral healthcare [43].

Building on this idea of maximizing existing resources, another opportunity lies in leveraging the existing dental workforce, particularly in contexts like India, where oral healthcare is predominantly delivered through the private sector [29]. Rather than viewing this reliance as a limitation, it can be reframed as a strategic advantage. Here, Japan offers a great example, where the majority of dental services are provided by private practitioners who operate under government contracts and are reimbursed through public funding [44]. In the Indian context, such an approach would transform a structural challenge into a scalable solution, advancing towards equitable oral healthcare through Public–Private partnerships.

Reframing existing constraints as opportunities in oral health policy reveals a pressing need for systemic reform, one that goes beyond resource optimization to fundamentally redefine care priorities. At the heart of this transformation is a paradigm shift from curative or operative to preventive care, a transition strongly advocated by the WHO [10] and echoed by participants in this study. Prioritizing prevention not only reorients how societies value oral health but also paves the way for sustainable outcomes, reduced long-term costs, and improved overall well-being [45]. Japan’s successful ‘8020 campaign’ which aimed to retain 20 natural teeth by the age of 80, is a population-level preventive strategy particularly relevant in aging societies, demonstrating how preventive care not only reduces the financial burden of untreated oral diseases, but also helps maintain oral health and masticatory function, contributing to the prevention of aspiration pneumonia and thereby preserving overall quality of life [44,46].

While advocacy for preventive dental care continues to grow globally, it is often accompanied by a recognition of persistent inadequacies in this area [25,27,44]. This de-prioritization of preventive care in oral health was also strongly echoed by the participants in the study, who emphasized the urgent need to restructure dental education and curricula to focus more on comprehensive, prevention-oriented services rather than predominantly treatment-based approaches. This concern resonates with findings from a European study, where dentists reported feeling inadequately trained in preventive care owing to the treatment-heavy focus of their education [47]. Evidence also suggests that the undergraduate curriculum in India is focused more on clinical skills and lesser importance is given to primary prevention [48]. Addressing this gap in training is essential to empower practitioners to deliver care that aligns with evolving public health priorities.

For preventive strategies to be truly effective, they must directly address the most prevalent and high-impact oral health issues like dental caries and tobacco-related conditions, where evidence shows targeted interventions can significantly improve public health outcomes and reduce long-term costs [5,49]. In this context, India has a high oral cancer prevalence within South Asia [6], highlighting the urgent need to direct resources toward prevention, early detection, and timely treatment of oral cancer.

To drive broader change, achieving the highest level of integration for oral health requires more than financial mechanisms; it demands embedding oral health within national health policies and primary healthcare systems. This means ensuring oral health is represented at every level, from legislative frameworks to field-level implementation, with a strong emphasis on its inclusion in primary healthcare and UHC, a vision that has gained global momentum after being advocated at the first WHO global oral health meeting, where the Bangkok declaration was adopted in November 2024 [4].

Several participants in the study acknowledged the well-established primary healthcare infrastructure in India, which they viewed as a strong foundation for integrating oral health services and expanding reach to the masses. They also appreciated the growing momentum around the National Oral Health Policy in recent times. Although still in its early stages, recent evidence [29] suggests India is positioned ahead of many neighbouring countries in policy development. This is particularly evident through its mandate to place dentists at every PHC, marking a foundational step towards systemic integration.

Despite notable policy advancements and the presence of a strong healthcare infrastructure, several implementation challenges continue to hinder the effective integration of oral health into primary care in India. These challenges span across systemic, operational, and workforce-related domains, revealing a gap between policy intent and ground-level realities. Perhaps the biggest gap that remains in this area concerns the uneven distribution of the dental workforce and retention in the rural areas, as suggested by study participants as well as the global evidence [39]. Although India has a favourable dentist-to-population ratio, rural and underserved areas continue to face severe shortages of dental professionals. Participants attributed this maldistribution to multiple factors, most notably inadequate rural infrastructure and a lack of essential amenities such as quality education, roads, and other facilities, which fail to attract dental graduates. This paradox is evident across the globe, including the US [50], Australia [31], South East Asia [29], and parts of Europe, such as Romania and Spain [33], where dental professionals prefer to establish their practices in urban settings.

To address this imbalance, the study participants proposed a series of actionable strategies, encompassing the effective use of existing resources, infrastructure development, educational reforms, and enhanced research efforts. A key recommendation was to leverage India’s network of over 300 dental institutes, a substantial yet underutilized resource, by establishing partnerships with nearby PHCs. These collaborations could provide essential manpower and services, thereby expanding oral health coverage in a sustainable manner and alleviating pressure on tertiary care facilities. In addition, participants emphasized the importance of improving rural infrastructure and offering both financial and non-financial incentives, including housing, educational support, and subsidized loans for private practitioners, to attract and retain dental professionals in underserved areas. Some participants also suggested that students from rural regions may be more inclined to establish their practice in such areas. Nonetheless, even for these students, returning to rural practice may depend on the availability of supportive measures such as assured income opportunities, rural infrastructural development, and continued avenues for career growth. Without addressing these practical concerns, the willingness of rural-background graduates alone may not be sufficient to ensure long-term retention.

A transformative reform proposed was the alignment of dental education with the goals of rural service delivery. Inspired by Thailand’s successful model [51], compulsory rural postings for dental graduates were suggested as a means to redistribute the workforce and expose young professionals to the realities of rural health challenges. Participants also stressed the need for broader curricular reforms, particularly to enhance clinical training and positively shape dental graduates’ professional attitudes and social values. This observation aligns with existing literature, which suggests that the current curriculum remains disproportionately focused on cognitive knowledge, with limited emphasis on developing psychomotor (clinical) skills and almost no attention to affective domains such as empathy, ethics, and social responsibility [48]. To bridge this gap, recent evidence emphasizes the need to transition towards a competency-based dental education model that integrates knowledge, clinical proficiency, and attitudinal development in a cohesive and well-rounded manner [48,52]. Complementing this, participants strongly advocated the integration of community-based learning into dental curricula to cultivate empathy and social responsibility among dental graduates. This approach finds support in the proposed Sevagram model [53,54], mentioned by a participant, which fosters long-term community engagement. It also aligns with global evidence highlighting the value of community-based learning in promoting professionalism and humanism in dental education [55]. Finally, participants emphasized the importance of targeted research to better understand the motivations and barriers influencing dental professionals’ practice choices. This aligns with the global priority in public oral health research, which highlights the importance of exploring the behavioural, social, and systemic determinants of oral health [56].

As an alternative and complementary solution to the shortage of dentists, participants also suggested integrating dental auxiliaries into the oral healthcare workforce to improve access in underserved areas. This model is supported by global evidence [33,57], where team-based dental care is employed and DHAPs have been utilized to increase access in underserved regions [31,58].

Although this qualitative study is deeply rooted in the Indian context, its insights transcend national boundaries and resonate with countries undergoing health system transitions across the globe. The systemic challenges identified, such as workforce maldistribution, policy fragmentation, and service inequality, mirror global patterns, making the findings relevant to broader efforts in oral health reform. By aligning with international priorities like integrating oral health into primary care, strengthening prevention, and exploring sustainable financing models, the proposed solutions in this study provide a globally adaptable framework. The emphasis on locally grounded yet globally informed strategies could help empower policymakers, educators, and health advocates to craft culturally sensitive and system-responsive interventions, thereby reinforcing the value of this study.

While the findings may potentially offer insights relevant beyond the Indian context, this study contributes to the field by centering on the voices of public health dentists, professionals working at the intersection of clinical delivery, community engagement, and policy implementation. Unlike previous research, this study captures the lived experiences and reflective viewpoints of those actively engaged in oral health service delivery and reform. Through this lens, the study identifies structural constraints while also bringing attention to emerging grassroots innovations and underutilized institutional resources. By linking local observations to broader policy directions, the study may enrich ongoing discussions by providing context-sensitive reflections that could inform future efforts toward more equitable and sustainable oral healthcare systems.

Being a qualitative study, this research is limited by its contextual boundaries and methodological scope; however, every effort was made to maintain methodological rigor. While the study offers rich insights into India’s oral health system through the eyes of public health dentists in academia with diverse regional representation, resource constraints prevented the inclusion of dentists currently stationed at PHCs. This may have resulted in limited representation from frontline care settings. Additionally, the sample reflects regional and institutional skewness, with overrepresentation from private colleges in southern India and minimal participation from the North-East, which may affect the transferability of findings to rural and underserved contexts. These limitations highlight areas for future research that could expand on the stakeholder base by including PHC dentists, patients, community health workers, and policymakers, thereby enabling a more comprehensive understanding of the system. Future studies should also consider more balanced sampling approaches, ensuring equitable regional and institutional representation. Additionally, longitudinal studies could explore the impact of proposed reforms over time, helping to evaluate implementation pathways and their outcomes. Despite these limitations, the study provides a critical, empirically grounded starting point for reimagining oral health systems in India and beyond.

## 5. Conclusions

This study offers a critical lens into the evolving landscape of oral healthcare in India, revealing not only the structural gaps and systemic inequities that persist but also the resilience and innovation emerging from within the system. The narratives of public health dentists illuminated a broader, more complex reality—one where educational shortcomings, policy inertia, regional disparities, and sociocultural barriers intersect with prospective solutions to shape access to oral healthcare. The findings underscore that addressing India’s oral disease burden requires a reimagining of how oral health is prioritized, delivered, and integrated into the broader public health agenda. By surfacing both the challenges and the promising grassroots initiatives already underway, this study calls for a paradigm shift toward a more equitable, community-rooted, and policy-supported oral healthcare system that is responsive to the diverse needs of India’s population. Collectively, these findings emphasize the need for a multipronged, context-sensitive approach that integrates educational reform, robust policy implementation, public–private collaboration, and community engagement to build a more inclusive, accessible, and sustainable oral healthcare system in India.

## Figures and Tables

**Table 1:** Interview Guide for qualitative data collection.

Oral healthcare landscape focused questions *	Could you tell me more about your association with the field of dentistry? How long have you been working specifically in public health dentistry? What kind of public health dentistry settings and activities do you engage in? How well are you and your organization able to meet your goals with respect to public health dentistry-related activities? What, in your opinion, is the present state of oral healthcare and related equity in India?
**DHAP-focused questions**	6. Share your opinion regarding the potential role of DHAPs in India? 7. How can DHAPs contribute to decreasing the oral disease burden in India? 8. Challenges in introducing training courses for dental nurses and dental therapists in India? 9. Employment opportunities for DHAPs following their training in India?
Oral healthcare landscape focused questions *	10. Any suggestions you would like to propose to manage the uneven distribution of the dental workforce in India? 11. Any plan to increase access to care in rural areas and address the oral health inequality in India? 12. Anything that you would like to share that we did not touch upon during this interview. Or any queries?

* These questions represent the focus of the qualitative study reported in this paper.

**Table 2:** Characteristics of the study participants.

Regions Across India	Number of Participants	Type of Dental College		Years of Academic Experience	
		Government	Private	≤10 years	>10 years
**North**	7	5	2	3	4
**South**	13	1	12	5	8
**East**	2	0	2	0	2
**West**	6	1	5	4	2
**Central**	2	0	2	0	2
**North-east**	1	1	0	1	0

## Data Availability

Data will be made available on reasonable request to the corresponding author in accordance with the Institutional Ethics Committee of Manipal College of Dental Sciences, Mangalore. However, the data cannot be made available freely for ethical considerations and the need to protect the confidentiality and privacy of the study participants.

## Supplementary Materials

The following supporting information can be downloaded at: https://www.mdpi.com/article/10.3390/ijerph22111741/s1, The SRQR checklist has been provided as Supplementary Material S1.

## Abbreviations

The following abbreviations are used in this manuscript:

NCD	Non-communicable disease
US	United States
USD	US dollars
WHO	World Health Organization
DALY	Disability-adjusted life-years
UHC	Universal health coverage
DHAP	Dental health associate professional
SRQR	Standards for Reporting Qualitative Research
NGO	Non-governmental organization
CSR	Corporate Social Responsibility
ASHA	Accredited social health activist
NOHP	National Oral Health Policy
BDS	Bachelor of Dental Surgery
PHC	Primary healthcare center
CHC	Community health center
PPP	Public–private partnership
UK	United Kingdom
OOP	Out-of-pocket
CAD	Canadian Dollars
OECD	Organization for Economic Cooperation and Development
NHS	National Health Service
